# Registration of presurgical MRI and histopathology images from radical prostatectomy via RAPSODI

**DOI:** 10.1002/mp.14337

**Published:** 2020-07-18

**Authors:** Mirabela Rusu, Wei Shao, Christian A. Kunder, Jeffrey B. Wang, Simon J. C. Soerensen, Nikola C. Teslovich, Rewa R. Sood, Leo C. Chen, Richard E. Fan, Pejman Ghanouni, James D. Brooks, Geoffrey A. Sonn

**Affiliations:** ^1^ Department of Radiology School of Medicine Stanford University Stanford CA 94305 USA; ^2^ Department of Pathology School of Medicine Stanford University Stanford CA 94305 USA; ^3^ School of Medicine Stanford University Stanford CA 94305 USA; ^4^ Department of Urology School of Medicine Stanford University Stanford CA 94305 USA; ^5^ Department of Urology Aarhus University Hospital Aarhus Denmark; ^6^ Department of Electrical Engineering Stanford University Stanford CA 94305 USA

**Keywords:** cancer labels, histopathology, MRI, prostate cancer, registration

## Abstract

**Purpose:**

Magnetic resonance imaging (MRI) has great potential to improve prostate cancer diagnosis; however, subtle differences between cancer and confounding conditions render prostate MRI interpretation challenging. The tissue collected from patients who undergo radical prostatectomy provides a unique opportunity to correlate histopathology images of the prostate with preoperative MRI to accurately map the extent of cancer from histopathology images onto MRI. We seek to develop an open‐source, easy‐to‐use platform to align presurgical MRI and histopathology images of resected prostates in patients who underwent radical prostatectomy to create accurate cancer labels on MRI.

**Methods:**

Here, we introduce *RA*diology *P*athology *S*patial *O*pen‐Source multi‐*D*imensional *I*ntegration (RAPSODI), the first open‐source framework for the registration of radiology and pathology images. RAPSODI relies on three steps. First, it creates a three‐dimensional (3D) reconstruction of the histopathology specimen as a digital representation of the tissue before gross sectioning. Second, RAPSODI registers corresponding histopathology and MRI slices. Third, the optimized transforms are applied to the cancer regions outlined on the histopathology images to project those labels onto the preoperative MRI.

**Results:**

We tested RAPSODI in a phantom study where we simulated various conditions, for example, tissue shrinkage during fixation. Our experiments showed that RAPSODI can reliably correct multiple artifacts. We also evaluated RAPSODI in 157 patients from three institutions that underwent radical prostatectomy and have very different pathology processing and scanning. RAPSODI was evaluated in 907 corresponding histpathology‐MRI slices and achieved a Dice coefficient of 0.97 ± 0.01 for the prostate, a Hausdorff distance of 1.99 ± 0.70 mm for the prostate boundary, a urethra deviation of 3.09 ± 1.45 mm, and a landmark deviation of 2.80 ± 0.59 mm between registered histopathology images and MRI.

**Conclusion:**

Our robust framework successfully mapped the extent of cancer from histopathology slices onto MRI providing labels from training machine learning methods to detect cancer on MRI.

## 1 INTRODUCTION

Despite advances in diagnosis and treatment, prostate cancer remains the second leading cause of cancer‐related death in American men.^1^ Overdiagnosis of low‐grade cancers that do not require treatment and the underdiagnosis of aggressive cancers are still major clinical dilemmas.^2^ magnetic resonance imaging (MRI) can help address these problems.^3^ Up to 50% of men with elevated PSA who would otherwise undergo biopsy, can safely avoid prostate biopsy when a pre‐biopsy MRI is normal. This approach reduces the overdiagnosis of low‐grade cancer and infectious complications of biopsy. However, this is only true when MRI is interpreted by world‐leading experts.^4^ In practice, the lack of widespread expertise and alarming levels of inter‐reader variation greatly reduce the potential of MRI to revolutionize prostate cancer diagnosis.^5^ Even when using the recommended Prostate Imaging‐Reporting and Data System (PIRADS),^6^ both false negatives and false positives are very common. MRI has yet to supplant biopsy, which is still required to confirm the presence and aggressiveness of prostate cancer.^7^

In men diagnosed with prostate cancer on biopsy, radical prostatectomy remains the most common treatment.^8^ The resected prostate provides a unique opportunity to correlate presurgical MRI with digitized histopathology images. Developing a large dataset of prostatectomy cases via automated registration of histopathology images and MRI, where cancer and Gleason grades are accurately mapped on MRI has two potentially transformative applications. First, such mappings can aid in improving existing MRI interpretation schemes that are still affected by many false positives and false negatives. Second, these mappings can facilitate the development of machine learning methods to identify prostate cancer on MRI by providing accurate cancer labels for model training and validation.

Although numerous approaches for the radiology‐pathology registration in the prostate have been introduced (see section “Prior Work” in the Suplementary Material), these approaches have not been widely adopted and have not been carefully tested by scientists outside the developer teams. Recent publications using histopathology images as a reference to improve MRI and automatically detect cancer^9^, ^10^, ^11^, ^12^, ^13^ still require manual approaches to align the histopathology to MR images; these approaches are labor‐intensive and subjective. The slow adoption of previous methods is due to the challenges associated with managing and registering the histopathology images and MRI, the lack of open source methods, and the time constraints associated with running these methods.

Specifically, the registration of histopathology images and prostate MRI has the following challenges. Histologic processing of the resected tissue causes artifacts, for example, deformations, shrinkage, and tissue ripping. Some of these artifacts (e.g., deformation and shrinking) can be corrected through registration, while others (e.g., tissue ripping) are challenging to correct and may result in discarding slices when such artifacts are major. Furthermore, our method and many others^14^, ^15^, ^16^ assume slice‐to‐slice correspondence between histopathology and MR images, which can be improved through the use of customized 3D printed molds based on preoperative MRI.^17^ However, this approach requires a change in clinical protocol that may not be present in the vast majority of institutions performing radical prostatectomy. Finally, the acquired data are different between the histopathology images and MRI. Histopathology images provide a discontinuous serial stack of colored images with a pixel size of 0.0005 mm and 4 μ*m* thickness, separated by roughly 5 mm spaces, while MRI has a typical resolution of 0.4 × 0.4 × 4.0 mm^3^.

Here, we introduce the *RA*diology *P*athology *S*patial *O*pen‐Source multi‐*D*imensional *I*ntegration (RAPSODI) framework for the registration of histopathology slices and preoperative MRI. RAPSODI includes a memory‐efficient registration methodology and a Graphical User Interface Plugin to 3D Slicer.^18^ Our registration approach relies on the 3D reconstruction of the histopathology specimen to create a digital representation of the tissue before gross sectioning. Next, RAPSODI registers corresponding histopathology and MRI slices. Finally, the optimized transforms are applied to the cancer regions outlined on the histopathology images to project those labels onto the preoperative MRI.

We evaluated our methodology using a digital phantom where we simulated various artifacts resulting from the histologic preparation of the excised tissue, for example, rotation of the tissue when mounting on the glass slide or tissue shrinkage. Moreover, we tested RAPSODI in 157 prostate cancer patients that underwent radical prostatectomy from three institutions.

Our approach makes the following contributions: (a) our registration methodology combines a 3D reconstruction of the histopathology specimen with 2D affine and deformable registration of the corresponding histopathology and MRI slices and was optimized for accurate alignment, (b) our approach was tested in a digital phantom where the ground truth is known as well as in the largest cohort considered to date in a radiology‐pathology registration study, and (c) to the best of our knowledge, we are the first to release the source code for the registration of histopathology and radiology images in the prostate, which is essential to test the reproducibility and robustness of the approach while allowing wide adoption.

## 2 MATERIALS AND METHODS

### 2.1 Notations

Let $M:R^{3}\to R$ be the T2‐weighted (T2w) MRI with a matrix size of *K* × *L* × *M*. Let $H:R^{3}\to R^{3}$ be the stack of histopathology slices obtained by stacking 2D histopathology slices, $H_{i}:R^{2}\to R^{3}$. H has dimensions *W* × *H* × *D*, where *D* represents the number of slices, while *W* and *H* are the width and height of the histopathology images. The index *i*, is used to indicate either an axial slice within the MRI volume or an image in the histopathology stack. $M^{\mathrm{Pr}}$ and $H^{\mathrm{Pr}}$ represent the prostate segmentation on MRI and histopathology images, respectively.

### 2.2 Data description

The study was approved by the Institutional Review Board (IRB) of Stanford University (protocol number: IRB‐44998, title: “Characterizing the Radiologic Appearance and Changes Induced by Diseases”). Informed consent was waived for this retrospective study, according to our IRB protocol. Our study includes *N*_1_ = 116 subjects from Stanford Hospital (Cohort C1), *N*_2_ = 16 patients from the “Prostate Fused MRI Pathology” collection,^19^ The Cancer Imaging Archive (Cohort C2, Table S3) and *N*_3_ = 25 patients from the “Prostate MRI” collection,^20^ The Cancer Imaging Archive (Cohort C3, Table S3). All subjects in our three cohorts underwent radical prostatectomy.

*MRI:* Multi‐parametric MRI exams were available for all patients from 3 Tesla MRI scanners from different manufacturers. Our study utilized the axial T2w MRI, which is acquired using a 2D Spin Echo protocol (Table S3). MRI exams in Cohort C1 were available predominately from GE scanners (GE Healthcare, Waukesha, WI, USA), with some exceptions from Siemens (Siemens Healthineers, Erlangen, Germany) and Philips (Phillips Healthcare, Amsterdam, Netherlands) and were acquired with external body array coils. The MRI exam for the patients in Cohorts C2^19^ and C3^20^ were acquired using an endorectal coil either by a Siemens scanner (Siemens Healthineers, Erlangen, Germany) or Phillips scanner (Phillips Healthcare, Amsterdam, Netherlands). We used only the T2w MRI for the registration as it provides the best soft tissue contrast and spatial resolution, capturing anatomic features that help the registration with the MRI, for example, the boundary between peripheral zone and central gland or benign prostatic hyperplasia nodules.

*Histopathology:* Following resection and fixation in formalin, the prostates in the Cohorts C1 and C3 were sectioned using a patient‐specific 3D printed mold built based on the presurgical MRI. The gross sections for the prostates in Cohort C2 were cut perpendicular to the urethra from the apex to the mid gland. Mounting of the 5 μm thick tissue on the glass slide can cause rotation as well as left‐right flipping. An expert indicated the gross rotation angle and whether the slice requires left‐right flipping. The whole‐mount slices (Cohorts C1 and C3) and quadrants (Cohort C2) were stained using Hematoxylin & Eosin (H&E) and digitized at 20x magnification (pixel size 0.5 μm) for Cohorts C1 and C2 and at low resolution for C3. Frozen sections are only rarely used during prostatectomy at our institution (Cohort C1). When it is performed, additional tissue is excised from tissue remaining in the body rather than from the prostate itself. Therefore, no artifacts from frozen section are present within the excised gland. Pseudo‐whole mounts were generated for the images in Cohort C2 by the dataset authors and were achieved by stitching adjacent quadrants as described in Ref. ^21^. These pseudo‐whole mounts were made available by the authors of the dataset and were used without any further processing for the registration with MRI by RAPSODI. The quadrants must be stitched to form pseudo‐whole mounts to achieve registration with the MRI slices.

*Cancer labels:* Our expert genitourinary pathologist (CK, 14 yr of experience), outlined the extent of cancer on the histopathology images in Cohort C1. The cancer regions for the histopathology images in Cohorts C2 and C3 were labeled at their source institutions. The authors of the Cohort C2 also provided the cancer labels relative to the MRI which were obtained using landmark‐based registration when aligning histopathology images and MRI.^21^ For the subjects in Cohort C3, cancer regions were available as sharpie outlines marked directly on the glass slide.

*Other labels:* The registration of histopathology and MR images relies on the prostate segmentation on both modalities and slice‐to‐slice correspondences between MRI and histopathology images. To evaluate the registration, we used corresponding anatomic landmarks. Segmentations of the prostate, urethra, and anatomic landmarks were initially performed by trainees (WS, JBW, SJSC, and JCT) with 6+ months experience in this task and were carefully reviewed by our experts (CK, PG — a body MR imaging radiologist with 14 yr of experience, MR — an image analysis expert with 9 yr of experience). Two hundred fifty‐seven matching anatomic landmarks, for example, benign prostate hyperplasia nodules, ejaculatory ducts, predominant features (fig. S3 Row 4), were picked on both histopathology and radiology images for a subset of 12 subjects from Cohort C1. Slice correspondences between MRI and histopathology images were initially identified by trainees (NCT, JBW, WS), and subsequently confirmed by experts (MR, GS – a urologic oncologist with 13 yr of experience), and validated by a multi‐disciplinary team.

*Labels:* Our expert pathologist (CK) outlined cancer on histopathology images, while our experts (MR, CK, JBW, SJCS, NCT, and PG) outlined the prostate on MRI and/or histopathology images. Two hundred fifty‐seven matching anatomic landmarks, for example, benign prostate hyperplasia nodules, ejaculatory ducts, predominant glands, were picked by prostate imaging experts (MR and NCT) on both histopathology and radiology images for a subset of 12 subjects. The urethra was outlined by our experts (MR and WS). Slice correspondences between the MRI and histopathology were identified by experts (MR, NCT, JBW, and WS) and confirmed by the urologist, and validated by a multi‐disciplinary team of radiologists, pathologists, and urologists.

### 2.3 Radiology‐pathology registration

**Fig. 1:**
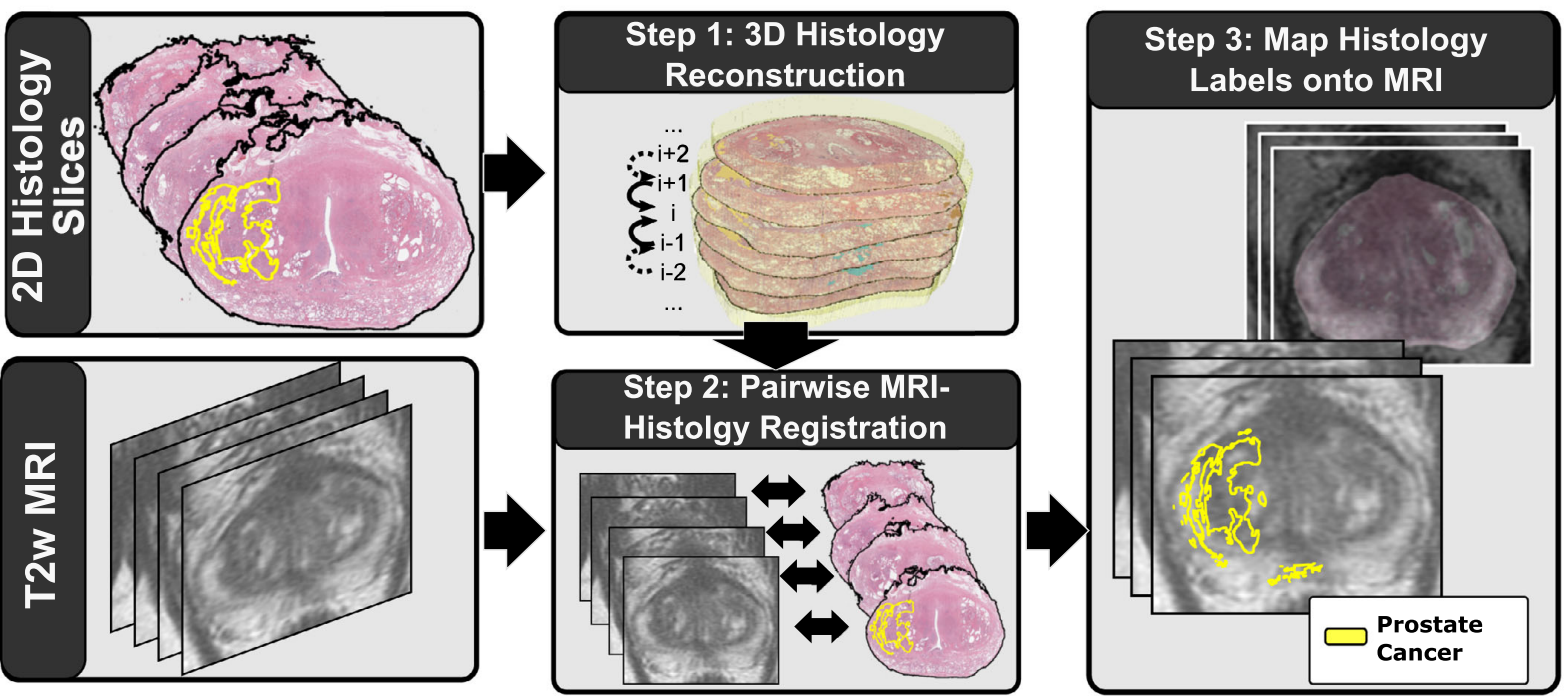
Summary of our approach. First, we align the serial histopathology slices relative to each other to reconstruct the three‐dimensional histopathology volume. Second, we register the histopathology slices relative to the T2w magnetic resonance imaging using rigid, affine, and deformable transforms. Finally, we map the extent of cancer from the histopathology images onto the radiology images. [Color figure can be viewed at wileyonlinelibrary.com]

Our approach is summarized in Fig. 1 and described below:

Preprocessing: We applied the prostate masks, $M^{\mathrm{Pr}}$ and $H^{\mathrm{Pr}}$ onto M and H, respectively, to exclude the structures outside the prostate from image registration. The gross rotation angles or left‐right flipping were applied.
3D Histopathology Reconstruction: We registered $H_{i}$ relative each other. We selected the middle slice $H_{i},i\;=\;\frac{M}{2}$ as fixed image and registered $H_{i-1}$ to $H_{i}$, $H_{i-2}$ with $H_{i-1}$, etc., as well as $H_{i+1}$ to $H_{i}$, $H_{i+2}$ with $H_{i+1}$, etc. With the exception of slice $i\;=\;\frac{M}{2}$, all histopathology images will have corresponding rigid transforms from the registration with the adjacent slice, $T_{\mathrm{Rig}}^{H}$.
2D Registration: We registered $M_{i}$ with $H_{i}$, for ∀*i* ∈ [1,*D*], by optimizing affine $T_{\mathrm{Aff}}^{M}$ and deformable $T_{\mathrm{Def}}^{M}$ transforms using a registration based on a multi‐resolution pyramid with three layers (shrinking factors: 16, 8, and 4; smoothing sigma: 4, 2, and 1 pixels). The affine registration only used the prostate masks during the optimization with sum of square differences as scoring function. The deformable registration was based on free‐form deformations^22^ with Mattes mutual information as a scoring function. The affine registration used a gradient descent optimizer with a learning rate of 0.01 and 250 iterations per resolution layer, while, the deformable registration employed a LBFGSB optimizer with ten iterations per resolution layer.
Mapping Cancer onto MRI: A composite transform of $T_{\mathrm{Rig}}^{H}$, $T_{\mathrm{Aff}}^{M}$ and $T_{\mathrm{Def}}^{M}$ is applied to deform the histopathology image as well as the cancer label and the anatomic landmarks into the coordinates of the T2w MRI.

Our approach was developed using the Insight Toolkit (ITK)^23^ and its Simple ITK API in python. The approach is available as a 3D Slicer python plugin^18^ (Fig. S4) or as a stand‐alone application to be run in batch mode (https://med.stanford.edu/rusulab/research.html). We measured the performance of the approach on an Intel i7‐8700 CPU, 3.70 GHz, 64 GB Memory Computer.

### 2.4 Digital phantom for radiology‐pathology registration

**Fig. 2:**
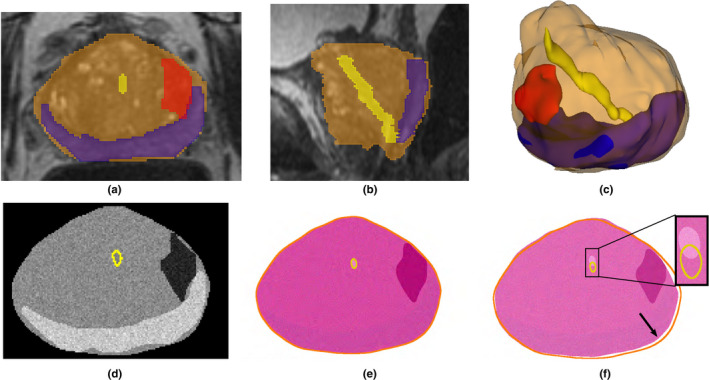
Radiology‐pathology digital phantom. The expert annotation of the prostate (orange), peripheral zone (blue), urethra (yellow), and cancer(red) on three‐dimensional (3D) T2 magnetic resonance imaging (MRI), shown in the (a) axial; (b) sagittal; (c) 3D views, were used to create a digital phantom of the prostate: (d) slice in the MRI phantom, (e) corresponding slice in the pathology phantom, and (f) imperfect corresponding slice that is 2 mm apart from (d) in the sagittal plane (yellow and orange are the outlines of the urethra and prostate(d)). Note the urethra misalignment (inset) and the border differences (arrow). [Color figure can be viewed at wileyonlinelibrary.com]

We created a digital phantom to assess the quality of the alignment when ground truth exists. The phantom is used to simulate artifacts known to affect histopathology sample preparation. We constructed the phantom by first outlining the prostate, peripheral zone, cancer, and urethra in a 3D T2w MRI [Figs. 2(a)–2(c)]. Then, we synthesized the phantom T2w MRI by filling the segmented regions with the average intensities from the input T2w image [Fig. 2(d)]. Moreover, we created the pathology phantom based on the histopathology images already registered to the T2w MRI (data not shown), by averaging their color intensities within the segmented regions [Figs. 2(e)–2(f)]. Our simulations included Gaussian noise on both the MRI and histopathology phantom slices.

Using the T2w and pathology phantoms, we tested three conditions: (a) the influence of the rotation angle when mounting the tissue slice on the glass slide, (b) the influence of shrinkage caused by fixation of the tissue during histology processing, and (c) the influence of imperfect slice correspondences between the MRI and histopathology slices, for example, Figs. 2(d) and 2(e) have a perfect correspondence, while Figs. 2(d) and 2(f) are 2 mm apart from each other in out‐of‐plain direction.

To evaluate RAPSODI, we used multiple conditions, for example, rotation, scaling, or imperfect slice correspondences. When a random rotation of *r* was assigned to the histopathology phantom, it resulted in applying a random angle ranging between −*r* and *r* to each slide and running ten experiments with different noise and random angle conditions to assess the mean and variance in performance. When rotations were applied alone, no translation or scaling was applied. When a shrinkage factor *s* is applied, all histopathology slices are shrunk by *s* relative to their original appearance. Moreover, we also apply a random translation of 5% in x or y directions. Thereby, the experiments that include rotation and shrinkage also include random translation, and when combined with the imperfect slice correspondences are close representations of real data.

### 2.5 Quantitative evaluation

The accuracy of the registration was evaluated using the Dice similarity coefficient, which assesses the overlap of the prostate outlines on T2w MRI and of the registered histopathology reconstruction:

(1) $$Dice\left(H,M\right)=\frac{1}{D}\sum_{i=1}^{D}\frac{2\times |H_{i}^{\mathrm{Pr}}\cap M_{i}^{\mathrm{Pr}}|}{\left|H_{i}^{\mathrm{Pr}}\left|+\right|M_{i}^{\mathrm{Pr}}\right|}$$

where *D* is the number of slices in the histopathology specimen, $H_{i}^{\mathrm{Pr}}$ represents the slice *i* in the prostate segmentation on histopathology, while $M_{i}^{\mathrm{Pr}}$ represents the slice *i* in the prostate segmentation on MRI.

Additionally, we evaluated the Hausdorff distance^24^ (a measurement of how far two subsets of a metric space are from each other) between the prostate boundary to assess boundary deviation after registration:

(2) $$\begin{array}{cc} Hausdorff^{\mathrm{Pr}}\left(H,M\right)= & \\ \frac{1}{D}\sum_{i=1}^{D}max\{sup_{h\in H_{i}^{\mathrm{Pr}}}\;inf_{m\in M_{i}^{\mathrm{Pr}}}d\left(H_{i}^{\mathrm{Pr}},M_{i}^{\mathrm{Pr}}\right), & \\ sup_{m\in M_{i}^{\mathrm{Pr}}}inf_{h\in H_{i}^{\mathrm{Pr}}}d\left(H_{i}^{\mathrm{Pr}},M_{i}^{\mathrm{Pr}}\right)\} \end{array}$$

where *sup* and *inf* represents the supremum and infimum operators, *D* represents the number of slices, and *i* is the slice index. The Hausdorff distance estimates the distance between the prostate borders (outlined on MRI and histopathology images), allowing us to estimate the registration error at the prostate boundary, without the need for corresponding landmarks.

Moreover, we evaluated the landmark distance:

(3) $$Dist\left(L^{H},L^{M}\right)=\frac{1}{X}\sum_{j=1}^{X}{\left|L_{j}^{H},L_{j}^{M}\right|}_{2}$$

where ${\left|.\right|}_{2}$ represents the Euclidean distance of the center of mass of the *j* ^*th*^ landmark $L_{j}^{H}$ on histopathology and center of mass of the *j* ^*th*^ landmark $L_{j}^{M}$ on MRI, while X represents the number of landmarks. Similarly, we computed the urethra distances on the slices where the urethra was visible on both MRI and histopathology images.

## 3 RESULTS

### 3.1 Phantom study

**Fig. 3:**
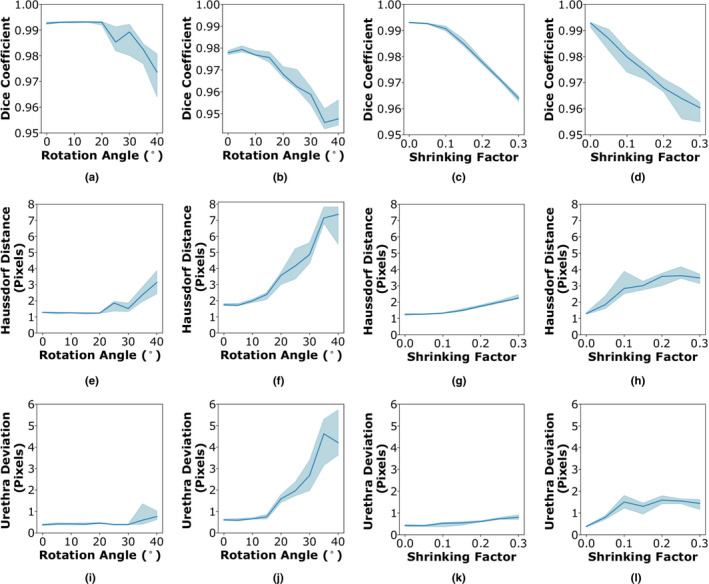
RAPSODI results for the registration of histopathology and T2w magnetic resonance imaging slices in the digital phantom in terms of (a)–(d) Dice coefficient, (e)–(h) Hausdorff distance, and (i)–(l) urethra deviation. (a, e, and i) Effect of the rotation (X‐axis) on the quantitative metrics when no shrinkage is applied; (b, f, and j) Constant 20% shrinkage is applied along with a randomly assigned rotations; (c, g,and k) Effect of tissue shrinkage, while no rotation was applied; (d, h, and l) A rotation angle of 20^∘^ is applied along with the shrinkage. [Color figure can be viewed at wileyonlinelibrary.com]

The phantom study is used to assess the average performance and variability of RAPSODI under conditions known to affect the tissue during the histopathology preparation. We ran our registration approach for 480 different conditions, to estimate the trends of the evaluation metrics as well as their variations. Figure 3 and Fig. S1 summarize our results in which we tested the effect of the rotation of histopathology slices while mounting on glass slides (range: 0–40^∘^), and the effect of shrinkage (range: 0–30%) when perfect slice correspondences exist between the histopathology and the MR images in the phantom. Our approach is able to perfectly recover rotation angles ranging between 0 and 20^∘^ or shrinkage of 0–10% when applied alone (Fig. 3 and Fig. S1), indicated by the perfect ∼ 1 Dice coefficient and the subpixel error. When combined, either using 20% shrinkage and random rotation or using 20^∘^ rotation and shrinkage, subpixel accuracy was observed for angles ranging between 0 and 15^∘^ or shrinkage of 0–5%. Beyond these conditions, RAPSODI is still able to recover induced rotation and shrinkage, yet with some errors as the starting conditions are far from the correct solution.

Moreover, the limitations of the registration may be observed when perfect correspondences are lacking between the histopathology and MRI slices (Fig. S1). Not surprisingly, the landmark and prostate border deviation are as large as four pixels (1.6 mm), as these features are not perfectly matching. Yet, we can observe the relative stability of the approach for rotations ranging 0–30^∘^ and shrinkage factors up to 30%, as the induced rotation and shrinkage are properly recovered.

### 3.2 Qualitative results

**Fig. 4:**
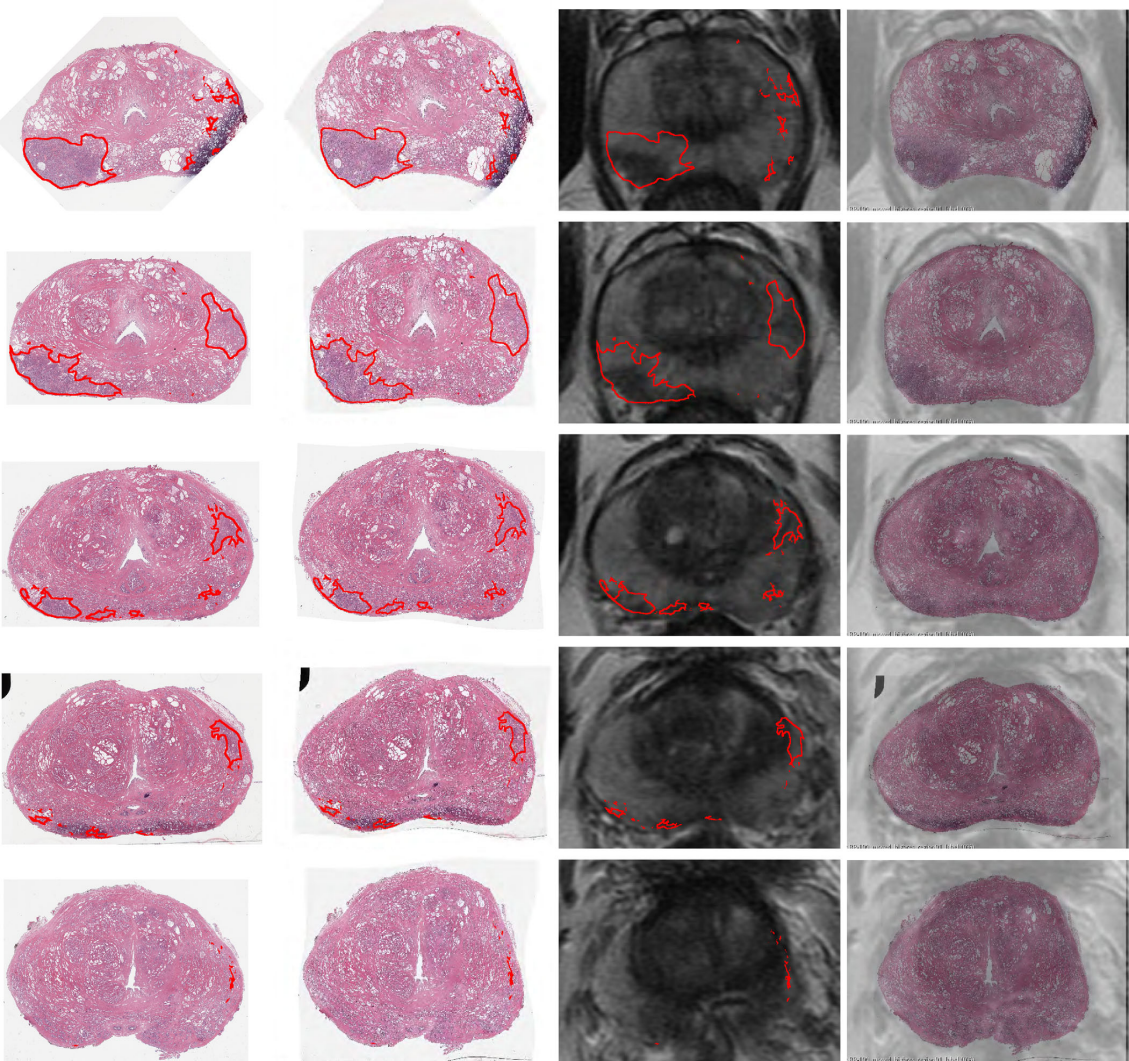
Qualitative results showing the registration for all the histopathology slices from apex to base in one subject in Cohort C1. (Column 1) Input histopathology slices with cancer outlines (red); (Column 2) histopathology slices registered to magnetic resonance imaging (MRI); (Column 3) corresponding T2w MRI with cancer outlines obtained via RAPSODI; (Column 4) overlay of the registered histopathology images and corresponding T2w MRI. [Color figure can be viewed at wileyonlinelibrary.com]

We applied RAPSODI to register the histopathology slices and T2w MRI in our radical prostatectomy cohorts of 157 patients. Figure 4 shows the qualitative results for a subject in Cohort C1 that had a Dice Coefficient of 0.98. Figure S2 shows the same slice as Fig. 4 Row 2, with progressive transparency from right to left and left to right to emphasize the alignment of the two modalities (see Movie S1). The qualitative and quantitative evaluation suggests that proper alignment was obtained.

The accurate registration allowed us to map the extent of two cancer foci with different Gleason groups^25^ (Fig. S2, yellow — Gleason group 1; green — Gleason Group 3). Although the higher grade cancer is visible on MRI, it appears smaller than the histopathology projected lesion, confirming previous work showing that MRI underestimates cancer size.^26^ The fusion enabled the mapping of the Gleason Group 1 cancer (yellow), which is not visible on MRI, and would have been otherwise difficult to outline.

Figure S3 shows a subject in Cohort C2 for which the alignment of the prostate achieved a Dice coefficient of 0.95 and a Hausdorff distance of 3.14 mm on the prostate boundary. The histopathology images (Fig. S3 Column 1) were registered with the MRI (Fig. S3 Column 3), and the cancer outline (red) was mapped onto MRI (Fig. S3 Columns 4 and 5) from the registered histopathology images (Fig. S3 Column 2). The public dataset includes the cancer annotation for this subject already mapped from the histopathology images onto MRI by the dataset authors.^21^ The cancer annotations obtained via RAPSODI overlaps well with the labels provided by the dataset authors (Fig. S3 Columns 5), with a Dice overlap of 0.53 and a Hausdorff Distance of 3.54 mm. The relatively low overlap indicated by the Dice coefficient may be accounted by the relatively small size of the tumor, and the misalignment of the regions in the apex slice (Fig. S3 Row 1).

### 3.3 Quantitative results

**Fig. 5:**
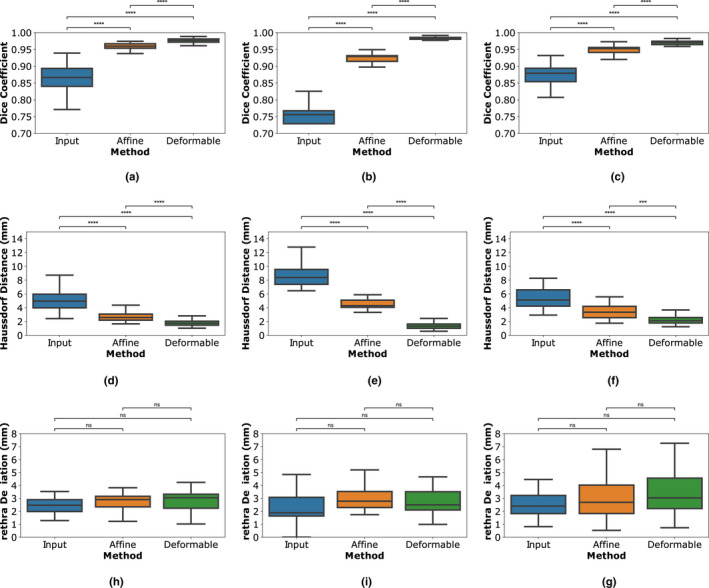
Quantitative evaluation of RAPSODI; (a)–(c) prostate Dice similarity coefficients, (d)–(f) Hausdorff distance on prostate boundary; (g–i) the urethra deviations. (a, d, and g) Cohort C1; (b, e, and h) Cohort C2; (c, f, and i) Cohort C3; ns — nonsignificant: 0.05 < *P* <= 1.00; ****P <= 0.0001 [Color figure can be viewed at wileyonlinelibrary.com]

An improvement in the alignment of the histopathology images and the T2w MRI can be observed across the different steps of our framework (Fig. 5). Statistically significant differences in Dice coefficients and Hausdorff distances were found between the input, and the results of the registration performed using RAPSODI (Mann‐Whitney test is statistically significant for *α* < 0.05). These statistically significant differences suggest that both affine and deformable registrations are required to facilitate an accurate alignment. The urethra [Figs. 5(g)–5(i)] and the landmark deviations showed no statistically significant differences.

The comparison of results across the three cohorts indicated that RAPSODI produces consistent results, with Dice coefficients of 0.96–0.98 on the prostate border, and Hausdorff distances averaging 1.84–2.57 mm (Table S2). The subjects in Cohorts C2 and C3 have MRIs acquired using an endorectal coil, which causes larger deformations of the prostate. Thereby, the input data and affine registration results show worse alignment, specifically in Cohort C2 compared to Cohort C1. However, similar metrics are evaluated after the deformable registration in RAPSODI, suggesting that our approach generalizes even for larger deformations, such as those induced by an endorectal coil.

An additional evaluation was possible in Cohort C2, since the authors of the dataset^19^ have provided the mapped cancer obtained via landmark‐based registration.^21^ Thereby, we compared the mapped cancer from RAPSODI with those provided by the dataset authors, and we observed a Dice coefficient of 0.55 ± 0.14 and deviation of 2.58 ± 1.34 mm computed on the center of mass. The relatively reduced alignment of the cancer labels may be attributed to the general misalignment error, which is within 3.1 mm inside the prostate and 2 mm on the prostate border. This misalignment can have a significant effect on the value of the Dice coefficient for regions of small size, such as the cancer, yet visually the alignment appears correct (Fig. S3 Column 5).

Due to the use of stitched histopathology images, and of endorectal coil MRI, larger deformations needed to be recovered when aligning the histopathology images to MRI in the patients in Cohort C2. The pseudo‐whole mounts can have stitching artifacts that are absent in the whole‐mount histopathology images. For example, the pseudo‐whole mount images are stretched in the anterior–posterior direction, for example, slice C1234 of patient aaa0054. The results presented here are obtained with the same parameters for all patients, from all cohorts, yet registration parameters such as scaling in the affine parameters can be modified, and the number of iterations increased further to allow recovery of large deformations (data not shown).

Unlike the subjects in the Cohorts C1 and C2, the histopathology images in the subjects in Cohort C3 were scanned at low resolution, having all slices scanned in the same image.^20^ This lower resolution has not affected the ability of RAPSODI to align the histopathology images to the MRI, indicating that our approach is robust to different histopathology scanning conditions.

## 4 DISCUSSION

Here, we introduced the RAPSODI framework that enables the registration of histopathology and MR images in the prostate. RAPSODI first reconstructs the histopathology volume, followed by a slice‐to‐slice alignment between the corresponding histopathology and T2w images. As shown in prior studies in the prostate^27^, ^28^ and other organs,^29^, ^30^, ^31^ the reconstruction ensures the consistent stacking of the histopathology slices relative to each other, independent of the MRI, which results in a better initialization in the registration with the MR images. Unlike prior studies^27^, ^28^, ^29^, ^30^, ^31^ that performed 3D registrations which are prone to overfitting due to a large number of degrees of freedom. RAPSODI performs the registration between MRI and corresponding histopathology images after reconstruction thus combining the benefits of the 2D registration to reduce the degrees of freedom with the 3D reconstruction to maintain 3D consistency.

Although numerous automated approaches for the registration of radiology and histopathology images have been proposed previously (see detailed discussion in the Supplementary material), manual registration approaches are still employed, even in recent publications.^9^, ^10^, ^11^, ^12^, ^13^ These manual approaches can generate subjective results and are tedious to use. They either rely on the user's expertise to identify and pick corresponding landmarks in the histopathology images and MRI^9^, ^10^, ^13^ or use cognitive alignments. Such cognitive alignments rely on a radiologist to directly outline the cancer region on MRI, with the help of a pathologist for radical prostatectomy cases,^11^, ^17^ yet such annotations are known to show smaller lesions that observed on pathology^26^ and are unable to capture MRI invisible lesions.

We first evaluated RAPSODI in a digital phantom and showed that our framework can recover the rotation of the histopathology slices resulting from glass slide mounting when these angles are within 15^∘^ from the correct solution and with tissue shrinkage up to 10%. Correcting for large rotation angles can be achieved prior to applying RAPSODI either using automated approaches, for example, by aligning the major axis of the data,^28^ or having an expert user indicating an angle, as was done in our study. The tissue shrinks during fixation with a factor that is outside our control. The affine transform helps identify the shrinkage factor, yet the accuracy of the registration declines as the initial conditions are further away from the optimal solution.

Prior automated registration of histopathology images with presurgical prostate MRI has been performed in proof‐of‐concept studies, which usually only include a small number of subjects, often  <  20 (Table S1), due to the use of intermediate images not routinely acquired for patients, for example, blockface pictures,^32^ ex vivo MRI^15^, ^16^, ^32^, ^33^ or external fiducials.^33^ RAPSODI uses clinical images and only relies on preoperative MRI and histopathology images for patients undergoing radical prostatectomy. RAPSODI was successfully used to register histopathology images with T2w MRI in the 157 subjects, achieving a prostate boundary error within 2 mm and an interior error within 3.1 mm. Through the use of prostate segmentation during registration, we emphasize the importance of the prostate border, resulting in a better alignment compared to the interior landmarks. Moreover, picking the landmarks used for evaluation can be challenging as we sought to capture 3+ landmarks/slice.

We acknowledge the following limitations of our approach. Although the registration approach is fully automated, similar to existing approaches, some manual interventions are needed to either segment the prostate on both MRI and histopathology images, to identify slice correspondences between the histopathology and T2w MRI or to correct the gross rotation of the histopathology slices. Yet, numerous techniques, for example,^34^, ^35^ have been developed to automatically segment the prostate on MRI, thereby the segmentation of the prostate can be automated (beyond the scope of the current paper). Unlike other approaches,^14^ RAPSODI does not rely on landmarks for the registration, but only uses them to evaluate the accuracy of registration.

The registration assumes that a slice‐to‐slice correspondence exists between the histopathology and MR images. While this is improved using 3D printed molds,^17^ which is routinely done for the patients undergoing radical prostatectomy at our institution, small slice misalignment is possible due to the shrinking of the prostate during fixation and shifting in the mold during slicing. Such misalignment is occasionally observed at the base and apex of the prostate. The digital phantom allowed us to study the effect of such misalignment and showed that a perfect registration alignment could not be obtained in this situation, yet the induced shrinkage and rotations are well recovered. Although our study includes patients for which the resected specimens were sectioned with (Cohorts C1 and C3) or without (Cohort C2) using 3D printed molds, we were unable to see a difference in alignment between the subjects in these different cohorts. The sectioning without 3D printed molds was likely done carefully by experienced histology technologists who were able to preserve the alignment with the MRI, even without using a 3D printed mold (Fig. S3).

The registration runtime for our approach is 0.5–4 min and depends on the resolution of histopathology images as well as the number of slices in each case. This runtime is limiting for a Graphical User Interface execution, yet it is acceptable when running the approach in batch mode. Deep learning methods^36^ can also be used to perform the registration with similar accuracy yet with a considerable reduction in runtime, but require such traditional registration methods to create the ground truth data.

Although MR image quality can vary, we were able to segment the prostate on MRI and perform the registration of histopathology and MR images in all cases. The affine registration is only influenced by the prostate segmentation, while the deformable registration is constrained to be rather stiff and not very elastic to limit possible overfitting. Thereby, we anticipate that the quality of the MRI will have little effect on our registration as long as the prostate boundary is clearly visible on the T2w MRI.

Our study includes 157 patients from three institutions, covering a wide range of image acquisition protocols: using MRI acquired via either surface or endorectal coils from three different manufacturers, various histopathology preparations (whole‐mount vs quadrants stitched in pseudo‐whole mounts), or different histopathology scanning resolution. To date, this work represents the largest study and the only study to evaluate registration in a digital histopathology‐MRI prostate phantom. Compared to previous approaches outlined in Table S1, our quantitative results place us close to the method by Kalavagunta et al.^14^ in terms of the Dice similarity coefficient. The latter approach relies on heavily annotated datasets that include the border of the transitional zone and the peripheral zone as well as other landmarks. Such landmarks are used during registration resulting in better landmark alignment, yet the approach is labor‐intensive and requires identifying matching landmarks in the pathology images and MRI, which can be challenging.

Through the registration of corresponding histopathology and MR images, RAPSODI allows for mapping the extent of cancer from histopathology onto MR images. This provides ground truth labels (“answer keys”) for the location and extent of cancer on MRI in patients who underwent radical prostatectomy. The availability of these “answer keys” has three clinical applications. First, they can be used as a training tool for radiologists. Second, they can be used to improve existing prostate MRI reading schemes, for example, PIRADS. Third, creating a large database of cases with MRI and ground truth cancer labels allows the training of machine learning models to automatically detect the extent of cancer on MRI, which in turn can assist radiologists when reading new prostate MRI studies. Models trained with labels mapped from histopathology images onto MRI have benefits compared to using labels manually outlined by radiologists, as they capture cancer borders more accurately and may be able to detect lesions on MRI that are invisible to radiologists.

## 5 CONCLUSIONS

RAPSODI aims to register histopathology and MR images with the goal of mapping cancer labels from histopathology images onto T2w MRI, thus creating careful and objective spatial labels on preoperative MRI. Such mapping may help develop advanced image analysis tools to predict prostate cancer reliably and its aggressiveness on MRI, can help improve current MRI interpretation schemes, and can help validate novel MRI protocols and other imaging techniques. Better imaging accompanied by better interpretation schemes can have a high impact on reducing overdiagnosis of low‐grade cancers, the underdiagnosis of aggressive cancers, and infectious complications of biopsy.

## CONFLICT OF INTEREST

The authors have no conflict to disclose.

## Supporting information


**Figure S1.** RAPSODI results for the registration of histopathology and T2w MRI slices in the digital phantom where an imperfect correspondence between the histopathology and T2w MRI slices exist (they are 2 mm apart from each other in the Sagittal and coronal planes, for example, Fig. 2d and 2f): (a and b) Dice coefficient; (c and d) Hausdorff distance; (e and f) urethra deviation. (a, c, and e) Experiment where only the rotation angle was varied between 0 and 40^∘^; (b, d, and f) The histopathology images were shrunk by 0–30% of the original size.
**Figure S2.** Overlay of registered histopathology and T2w images (same as slice as shown in Fig. 4 Raw 2). Histopathology shown with a progressive transparency from (a) right‐left, and (b) left‐right with cancer outlines (green — Gleason Group 3, yellow — Gleason group 1^25^).
**Figure S3.** Qualitative results showing the registration for all the histopathology slices from apex to base in subject aaa0059 from Cohort C2. (Column 1) Input histopathology slices with cancer outlines (red); (Column 2) Histopathology slices registered to MRI; (Column 3) Overlay of the registered histopathology and corresponding T2w MRI with histopathology images shown transparent. (Column 4) Corresponding T2w MRI with cancer outlines obtained via RAPSODI (red) or provided by dataset authors (blue); (Column 5) Close‐up into the cancer region with outlines shown at the same resolution as the T2w MRI. Asterisk (*) in row 4 indicates predominant features seen on both histopathology images and MRI that could be used as landmark to assess the registration.
**Figure S4.** Slicer Interface.
**Table S1.** Summary of previous approaches (not exhaustive). We excluded publications with <2 subjects, only synthetic data, or manually intensive approaches. All summarized methods require as input the in vivo presurgical T2 weighted MRI, digitized serial histopathology images, and the segmentation of the prostate on MRI and histopathology images; Additional input requirements are listed here; Abbreviations: TPS — Thin Plate Spline; NA — Not available
**Table S2.** Quantitative results for the three cohorts and aggregated for all subjects in our study.
**Table S3.** Data Summary: Abbreviations: T2‐weighted MRI (T2w), hematoxylin & eosin (H&E), relaxation time (TR), Echo Time (TE) ; MRI matrix size: *K* × *L* × *M*, histology matrix size: W × H, * estimated, pseudo‐whole mount: stitched adjacent quadrants; # : number; Pr: prostate, Lm: landmarks, Ure: urethra, Ca: cancer
