# Supplementary material for: Registration of presurgical MRI and histopathology images from radical prostatectomy via RAPSODI
Source: Med Phys. 2020 Jul 18;47(9):4177–88. doi: 10.1002/mp.14337 (PMC7586964; doi:10.1002/mp.14337)
Supplement: Supplementary file 1 — Figure S1. RAPSODI results for the registration of histopathology and T2w MRI slices in the digital phantom where an imperfect correspondence between the histopathology and T2w MRI slices exist (they are 2 mm apart from each other in the Sagittal and coronal planes, for example, Fig. 2d and 2f): (a and b) Dice coefficient; (c and d) Hausdorff distance; (e and f) urethra deviation. (a, c, and e) Experiment where only the rotation angle was varied between 0 and 40∘; (b, d, and f) The histopathology images were shrunk by 0–30% of the original size. Figure S2. Overlay of registered histopathology and T2w images (same as slice as shown in Fig. 4 Raw 2). Histopathology shown with a progressive transparency from (a) right‐left, and (b) left‐right with cancer outlines (green — Gleason Group 3, yellow — Gleason group 125). Figure S3. Qualitative results showing the registration for all the histopathology slices from apex to base in subject aaa0059 from Cohort C2. (Column 1) Input histopathology slices with cancer outlines (red); (Column 2) Histopathology slices registered to MRI; (Column 3) Overlay of the registered histopathology and corresponding T2w MRI with histopathology images shown transparent. (Column 4) Corresponding T2w MRI with cancer outlines obtained via RAPSODI (red) or provided by dataset authors (blue); (Column 5) Close‐up into the cancer region with outlines shown at the same resolution as the T2w MRI. Asterisk (*) in row 4 indicates predominant features seen on both histopathology images and MRI that could be used as landmark to assess the registration. Figure S4. Slicer Interface. Table S1. Summary of previous approaches (not exhaustive). We excluded publications with <2 subjects, only synthetic data, or manually intensive approaches. All summarized methods require as input the in vivo presurgical T2 weighted MRI, digitized serial histopathology images, and the segmentation of the prostate on MRI and histopathology images; Additional input requirements are [file MP-47-4177-s001.pdf]

Registration of pre-surgical MRI and histopathology images from radical prostatectomy via RAPSODI
Rusu et. al.

ADDITIONAL INFORMATION

Prior Work

Although numerous automated approaches for the registration of radiology and histopathology images have been developed, manual approaches are still employed, even in recent publications [1–5]. Some manual or semi-automatic approaches utilize landmark-based registration approaches, either alone [1, 2, 5] or in combination with automated registration steps [2, 6]. These approaches are labor-intensive and require the human operator to possess expertise in both MRI and histopathology, and necessitate identification of corresponding landmarks on both modalities. Other approaches [3, 7] employ cognitive alignment in which a radiologist with the help of a pathologist directly outlines the cancer region on MRI considering the histopathology images as reference. Such methods are tedious to apply and may be prone to underestimating the dimensions of the lesion [8], while MRI invisible lesions are hard if not impossible to outline and thereby they are often omitted from follow-up analysis. A few approaches use interactive image transformations [9], in which a user indicates scaling, rotations, and translations to be applied to the images. Such approaches are also tedious to utilize and require extensive knowledge in both radiology and pathology of the prostate.

The automated registration of histopathology images with pre-surgical prostate MRI has been performed in proof-of-concept studies, which usually only include a small number of subjects, often < 20 (TABLE S1). Most approaches assume a slice-to-slice correspondence between the histopathology images and T2 weighted (T2w) MRI slices. Some partial correspondence commonly results from the gross sectioning of the prostate in histologic preparation, which is done perpendicular to the urethra in the apical part of the prostate. In some studies, more advanced methods have been introduced to enforce such correspondences. For example, three dimensional (3D) printed patient-specific molds [7] have been used [6, 10, 11] to help preserve the correspondences during tissue sectioning. Some studies additionally included blockface picture [12], ex vivo MRI [6, 11–13] or external fiducials [13] to help improve the accuracy of the registration. These approaches required modifications of the clinical protocols, usually resulting in only a small number of subjects recruited for such research studies.

Once correspondences between the histopathology images and T2w MRI are identified, their registration can still be challenging, partially due to the artifacts induced by the tissue preparation. Textural features [14, 15] have been proposed, yet they may be cumbersome to use due to the high-dimensional scoring function optimization and the choice of textural features. Other approaches rely solely on image intensity to drive the deformable alignment [16, 17], but require accurate affine alignment prior to the deformable registration.

Previous work in the lung [18, 19], breast [20] or prostate [16, 17], has relied on approaches that reconstruct the sequential histopathology slices and created a 3D volume representing the histopathology specimen prior to sectioning, which facilitates the spatial registration with the 3D volumetric MRI and alleviates the need for slice correspondences. However, these methods are prone to overfitting the histopathology reconstruction due to a large number of degrees of freedom and may suffer from partial volume effects due to thick MRI slices and the histopathology slice spacing.

SUPPLEMENTARY TABLES

TABLE S1: Summary of previous approaches (not exhaustive). We excluded publications with <2 subjects [21], only synthetic data [22], or manually intensive approaches [9]. All summarized methods require as input the in vivo pre-surgical T2 weighted MRI, digitized serial histopathology images, and the segmentation of the prostate on MRI and histopathology images; Additional input requirements are listed here; Abbreviations: TPS - Thin Plate Spline; NA - Not available

Publication	Subject #	Approach	Additional Input	Dice Coef.	Landmark Error (mm)
Park 2008 [12]	2	3D reconstruction + affine and TPS registration	block face picture, ex vivo MRI	NA	3-3.74
Chappelow 2011[14]	25	Feature Based Mutual Information + BSpline	-	NA	NA
Ward 2012 [13]	13	2D Affine + TPS Registration	Strand-shaped fiducials, Ex vivo MRI	NA	1.1
Kalavagunta 2014 [10]	35	Local affine registration	Internal landmarks, 3D Printed Molds	0.99	1.54±0.64
Reynolds 2015 [6]	6	2D TPS registration + deformable registration	Control Points, ex vivo MRI, sectioning box	0.93	3.3
Li 2017 [15]	19	Multi-Scale Representation + deformable registration	-	0.96±0.01	2.96±0.76
Losnegard 2018 [16]	12	3D histopathology reconstruction, 3D affine and deformable registration	-	0.94	5.4
Wu 2019 [11]	17	2D Rigid, TPS Registration (automatic landmarks)	ex vivo MRI, 3D printed molds	0.87±0.04	2.0±0.5
Rusu 2019 [17]	15	3D histopathology reconstruction, 2D Affine+Deformable	3D printed Molds	0.94±0.02	1.11±0.34

TABLE S2: Quantitative results for the three cohorts and aggregated for all subjects in our study.

Cohort	Dice Prostate	Haussdorff Distance (mm)	Urethra Deviation (mm)	Landmarks Deviation (mm)	Dice Cancer
C1	0.98±0.01	1.84±0.54	2.74±0.85	2.80±0.59	-
C2	0.96±0.01	2.57±1.05	3.13±1.25	-	0.55±0.14
C3	0.97±0.01	2.35±0.85	3.52±2.04	-	-
All	0.97±0.01	1.99±0.71	3.09±1.45	2.80±0.59	0.55±0.14

SUPPLEMENTARY FIGURES

TABLE S3: Data Summary: Abbreviations: T2-weighted MRI (T2w), Hematoxylin & Eosin (H&E), Relaxation Time (TR), Echo Time (TE) ; MRI Matrix Size: $K \times L \times M$, Histology Matrix Size: W×H, * estimated, pseudo-whole mount: stitched adjacent quadrants; # : number; Pr: Prostate, Lm: Landmarks, Ure: Urethra, Ca: Cancer

		Cohort C1	Cohort C2	Cohort C3
Cohort	Data Source	Internal	Public [23]	Public [24]
	Subject Number	116	16	25
	Number of slice	759	65	83
MRI	Manufacturer	GE	Siemens	Philips
	Coil type	Surface	Endorectal	Endorectal
	Sequence	T2w	T2w	T2w
	TR (s)	3.9-6.3	3.7-7.0	8.9
	TE (ms)	122-130	107	120
	Matrix Size: K,L	256-512	320	512
	Matrix Size: M	20-44	21-31	26
	Pixel Spacing (mm)	0.27-0.94	0.41-0.43	0.27
	Distance Between Slices (mm)	3.0-5.2	4	3
	Annotations	Pr, Ure, Lm	Pr, Ca, Ure	Pr, Ure
Histopathology	Stain	H&E	H&E	H&E
	Type	whole-mount	pseudo-whole mount	whole-mount; Low-res
	Matrix Size: W,L	1572-7556	2368-6324	360-2401
	Pixel Spacing (mm)	0.008,0.016	0.007*	0.021*
	Use 3D printed Molds	Yes	No	Yes
	Distance Between Slices (mm)	Same as MRI	Same as MRI	Same as MRI
	Annotations	Pr, Lm, Ure, Ca	Pr, Ure, Ca	Pr, Ure, Ca

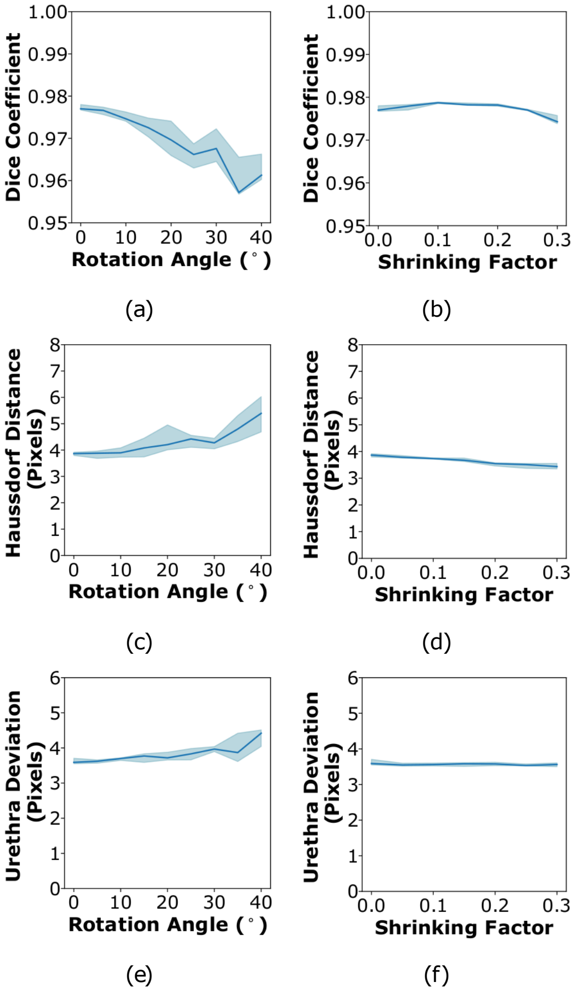

FIG. S1: RAPSODI results for the registration of histopathology and T2w MRI slices in the digital phantom where an imperfect correspondence between the histopathology and T2w MRI slices exist (they are 2 mm apart from each other in the Sagittal and coronal planes, e.g., Figures 2d,2f): (a-b) Dice Coefficient; (c-d) Hausdorff Distance; (e-f) Urethra Deviation. (a,c,e) Experiment where only the rotation angle was varied between 0-40°; (b,d,f) The histopathology images were shrunk by 0-30% of the original size.

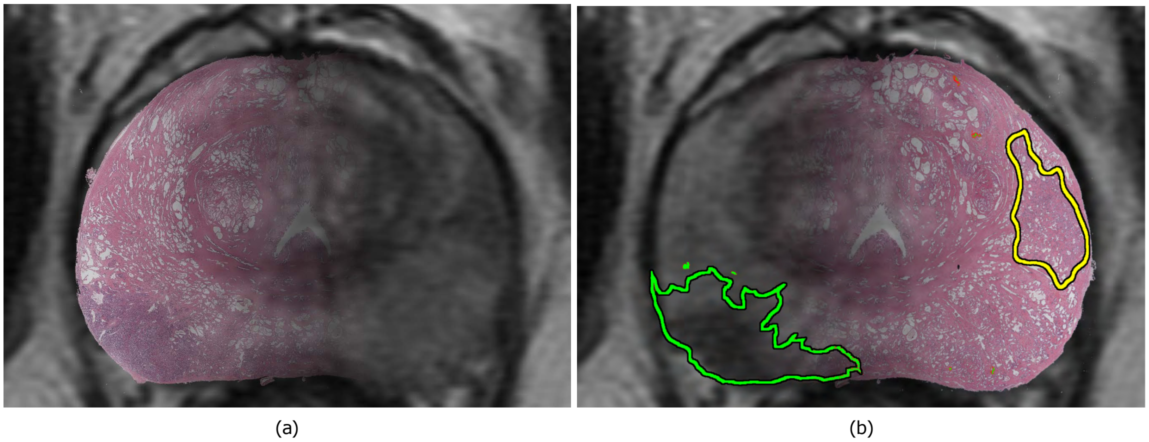

FIG. S2: Overlay of registered histopathology and T2w images (same as slice as shown in FIG. 4 Raw 2). Histopathology shown with a progressive transparency from (a) right-left, and (b) left-right with cancer outlines (green – Gleason Group 3, yellow-Gleason group 1 [25]).

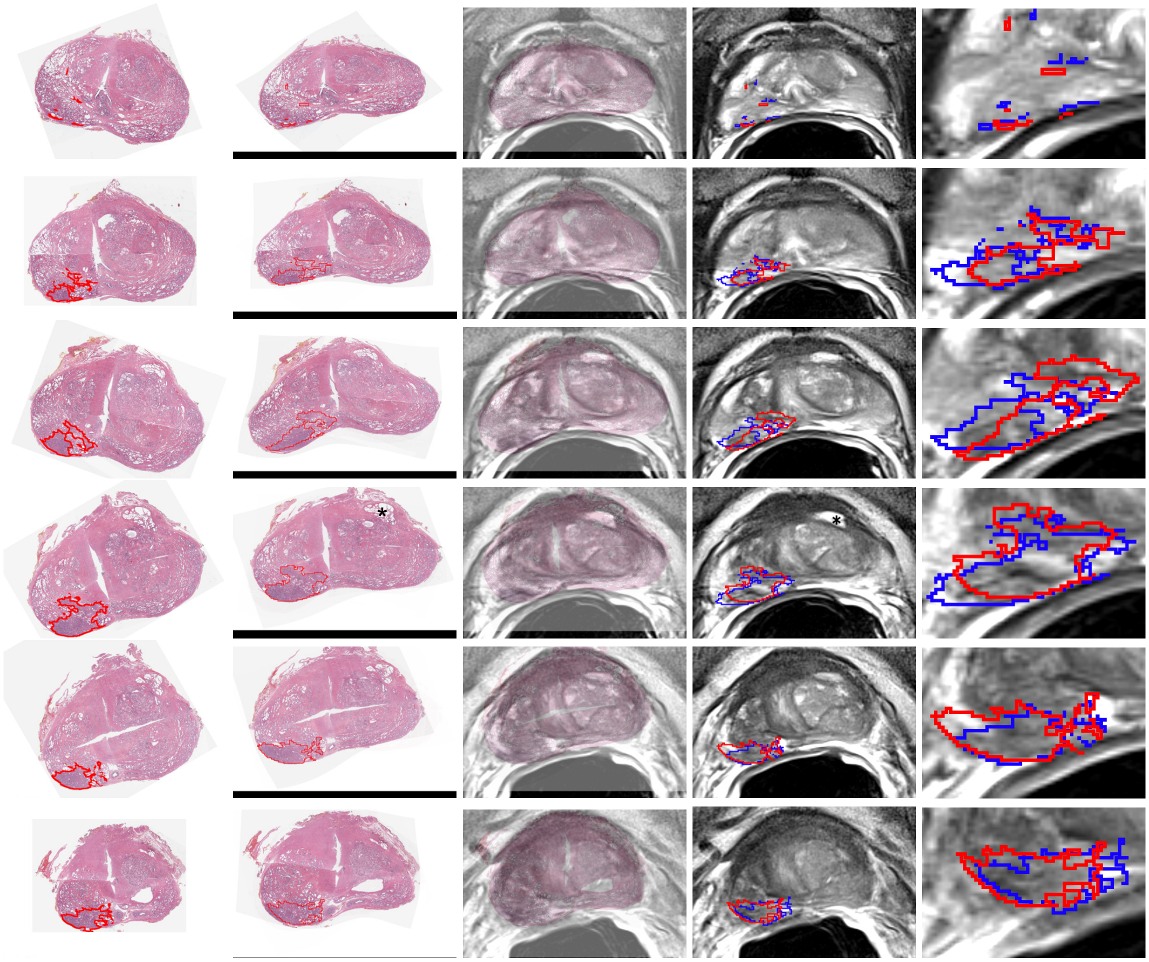

FIG. S3: Qualitative results showing the registration for all the histopathology slices from apex to base in subject aaa0059 from Cohort C2. (Column 1) Input histopathology slices with cancer outlines (red); (Column 2) Histopathology slices registered to MRI; (Column 3) Overlay of the registered histopathology and corresponding T2w MRI with histopathology images shown transparent. (Column 4) Corresponding T2w MRI with cancer outlines obtained via RAPSODI (red) or provided by dataset authors (blue); (Column 5) Closeup into the cancer region with outlines shown at the same resolution as the T2w MRI. Asterisk (*) in row 4 indicates predominant features seen on both histopathology images and MRI that could be used as landmark to assess the registration.

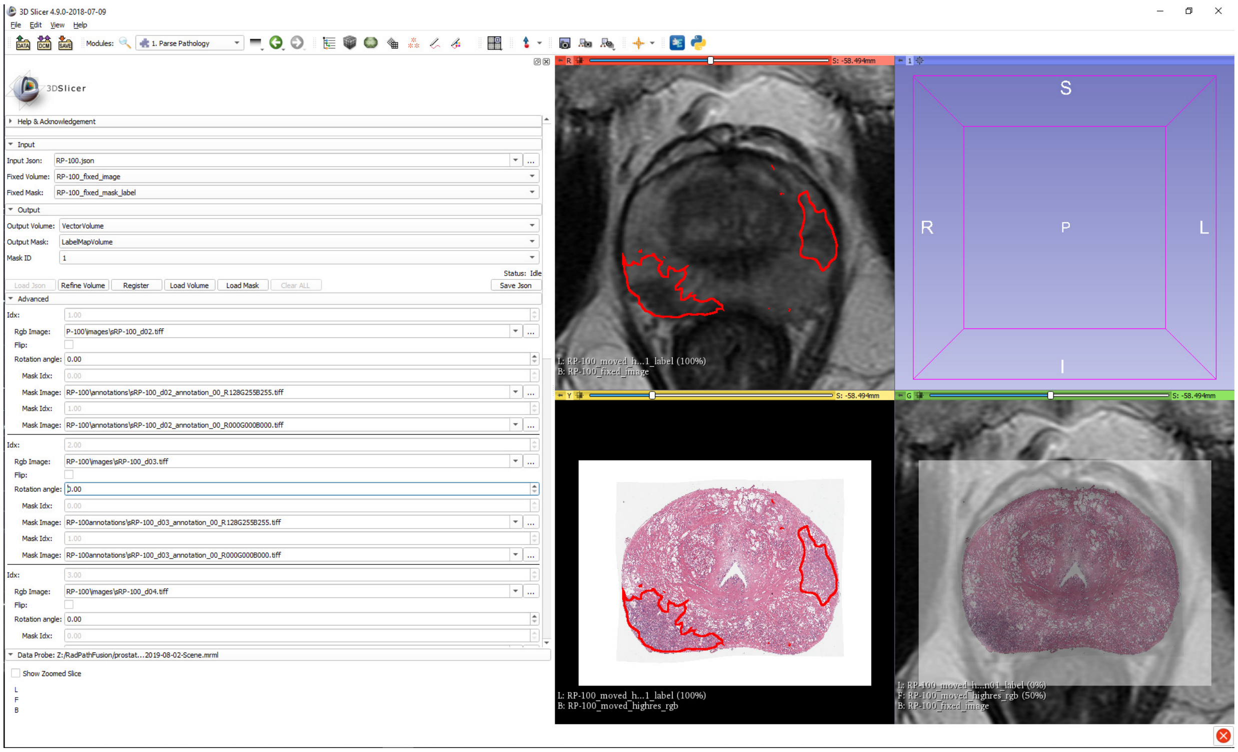

FIG. S4: Slicer Interface

[1] Gregory Penzias, Asha Singanamalli, Robin Elliott, Jay Gollamudi, Natalie Shih, Michael Feldman, Phillip D. Stricker, Warick Delprado, Sarita Tiwari, Maret Böhm, Anne-Maree Haynes, Lee Ponsky, Pingfu Fu, Pallavi Tiwari, Satish Viswanath, and Anant Madabhushi. Identifying the morphologic basis for radiomic features in distinguishing different Gleason grades of prostate cancer on MRI: Preliminary findings. *PloS One*, 13(8):e0200730, 2018.

[2] Sarah L. Hurrell, Sean D. McGarry, Amy Kaczmarowski, Kenneth A. Iczkowski, Kenneth Jacobsohn, Mark D. Hohenwalter, William A. Hall, William A. See, Anjishnu Banerjee, David K. Charles, Marja T. Nevalainen, Alexander C. Mackinnon, and Peter S. LaViolette. Optimized b-value selection for the discrimination of prostate cancer grades, including the cribriform pattern, using diffusion weighted imaging. *Journal of Medical Imaging (Bellingham, Wash.)*, 5(1):011004, 2018.

[3] Yohan Sumathipala, Nathan Lay, Baris Turkbey, Clayton Smith, Peter L. Choyke, and Ronald M. Summers. Prostate cancer detection from multi-institution multiparametric MRIs using deep convolutional neural networks. *Journal of Medical Imaging (Bellingham, Wash.)*, 5(4):044507, October 2018.

[4] Ruiming Cao, Amirhossein Mohammadian Bajgiran, Sohrab Afshari Mirak, Sepideh Shakeri, Xinran Zhong, Dieter Enzmann, Steven Raman, and Kyunghyun Sung. Joint Prostate Cancer Detection and Gleason Score Prediction in mp-MRI via FocalNet. *IEEE Transactions on Medical Imaging*, 38(11):2496–2506, November 2019. Conference Name: IEEE Transactions on Medical Imaging.

[5] Hayley M. Reynolds, Scott Williams, Price Jackson, Catherine Mitchell, Michael S. Hofman, Rodney J. Hicks, Declan G. Murphy, and Annette Haworth. Voxel-wise correlation of positron emission tomography/computed tomography with multiparametric magnetic resonance imaging and histology of the prostate using a sophisticated registration framework. *BJU International*, 123(6):1020–1030, June 2019.

[6] H. M. Reynolds, S. Williams, A. Zhang, R. Chakravorty, D. Rawlinson, C. S. Ong, M. Esteva, C. Mitchell, B. Parameswaran, M. Finnegan, G. Liney, and A. Haworth. Development of a registration framework to validate MRI with histology for prostate focal therapy. *Medical Physics*, 42(12):7078–7089, December 2015.

[7] Baris Turkbey, Haresh Mani, Vijay Shah, Ardeshir R. Rastinehad, Marcelino Bernardo, Thomas Pohida, Yuxi Pang, Dagane Daar, Compton Benjamin, Yolanda L. McKinney, Hari Trivedi, Celene Chua, Gennady Bratslavsky, Joanna H. Shih, W. Marston Linehan, Maria J. Merino, Peter L. Choyke, and Peter A. Pinto. Multiparametric 3 T prostate magnetic resonance imaging to detect cancer: histopathological correlation using prostatectomy specimens processed in customized magnetic resonance imaging based molds. *The Journal of Urology*, 186(5):1818–24, November 2011.

[8] Alan Priester, Shyam Natarajan, Pooria Khoshnoodi, Daniel J. Margolis, Steven S. Raman, Robert E. Reiter, Jiaoti Huang, Warren Grundfest, and Leonard S. Marks. Magnetic Resonance Imaging Underestimation of Prostate Cancer Geometry: Use of Patient Specific Molds to Correlate Images with Whole Mount Pathology. *The Journal of Urology*, 197(2):320–326, February 2017.

[9] Daniel N. Costa, Yonatan Chatzinoff, Niccolo M. Passoni, Payal Kapur, Claus G. Roehrborn, Yin Xi, Neil M. Rofsky, Jose Torrealba, Franto Francis, Cecil Futch, Phyllis Hagens, Hollis Notgrass, Susana Otero-Muinelo, Ivan Pedrosa, and Rajiv Chopra. Improved Magnetic Resonance Imaging-Pathology Correlation With Imaging-Derived, 3 D-Printed, Patient-Specific Whole-Mount Molds of the Prostate. *Investigative Radiology*, 52(9):507–513, 2017.

[10] Chaitanya Kalavagunta, Xiangmin Zhou, Stephen C. Schmechel, and Gregory J. Metzger. Registration of in vivo prostate MRI and pseudo-whole mount histology using Local Affine Transformations guided by Internal Structures (LATIS). *Journal of Magnetic Resonance Imaging*, 41(4):1104–1114, 2015.

[11] Holden H. Wu, Alan Priester, Pooria Khoshnoodi, Zhaohuan Zhang, Sepideh Shakeri, Sohrab Afshari Mirak, Nazanin H. Asvadi, Preeti Ahuja, Kyunghyun Sung, Shyam Natarajan, Anthony Sisk, Robert Reiter, Steven Raman, and Dieter Enzmann. A system using patient-specific 3 D-printed molds to spatially align in vivo MRI with ex vivo MRI and whole-mount histopathology for prostate cancer research. *Journal of magnetic resonance imaging: JMRI*, 49(1):270–279, January 2019.

[12] Hyunjin Park, Morand R. Piert, Asra Khan, Rajal Shah, Hero Hussain, Javed Siddiqui, Thomas L. Chenevert, and Charles R. Meyer. Registration methodology for histological sections and in vivo imaging of human prostate. *Academic Radiology*, 15(8):1027–1039, August 2008.

[13] Aaron D. Ward, Cathie Crukley, Charles A. McKenzie, Jacques Montreuil, Eli Gibson, Cesare Romagnoli, Jose A. Gomez, Madeleine Moussa, Joseph Chin, Glenn Bauman, and Aaron Fenster. Prostate: registration of digital histopathologic images to in vivo MR images acquired by using endorectal receive coil. *Radiology*, 263(3):856–864, June 2012.

[14] Jonathan Chappelow, B. Nicolas Bloch, Neil Rofsky, Elizabeth Genega, Robert Lenkinski, William DeWolf, and Anant Madabhushi. Elastic registration of multimodal prostate MRI and histology via multiattribute combined mutual information. *Medical Physics*, 38(4):2005–2018, April 2011.

[15] Lin Li, Shivani Pahwa, Gregory Penzias, Mirabela Rusu, Jay Gollamudi, Satish Viswanath, and Anant Madabhushi. Co-Registration of ex vivo Surgical Histopathology and in vivo T2 weighted MRI of the Prostate via multi-scale spectral embedding representation. *Scientific Reports*, 7(1):8717, August 2017.

[16] Are Losnegård, Lars Reisæter, Ole J. Halvorsen, Christian Beisland, Aurea Castilho, Ludvig P. Muren, Jarle Rørvik, and Arvid Lundervold. Intensity-based volumetric registration of magnetic resonance images and whole-mount sections of the prostate. *Computerized Medical Imaging and Graphics: The Official Journal of the Computerized Medical Imaging Society*, 63:24–30, January 2018.

[17] Mirabela Rusu, Christian Kunder, Richard Fan, Pejman Ghanouni, Robert West, Geoffrey Sonn, and James Brooks. Framework for the co-registration of MRI and histology images in prostate cancer patients with radical prostatectomy. In *Medical Imaging 2019: Image Processing*, volume 10949, page 109491P. International Society for Optics and Photonics, March 2019.

[18] Mirabela Rusu, Thea Golden, Haibo Wang, Andrew Gow, and Anant Madabhushi. Framework for 3 D histologic reconstruction and fusion with in vivo MRI: Preliminary results of characterizing pulmonary inflammation in a mouse model. *Medical Physics*, 42(8):4822–4832, August 2015.

[19] Mirabela Rusu, Prabhakar Rajiah, Robert Gilkeson, Michael Yang, Christopher Donatelli, Rajat Thawani, Frank J. Jacono, Philip Linden, and Anant Madabhushi. Co-registration of pre-operative CT with ex vivo surgically excised ground glass nodules to define spatial extent of invasive adenocarcinoma on in vivo imaging: a proof-of-concept study. *European Radiology*, 27(10):4209–4217, October 2017.

[20] Mirabela Rusu, Bruce Daniel, and Robert West. Spatial integration of radiology and pathology images to characterize breast cancer aggressiveness on pre-surgical MRI. In *SPIE Medical Imaging*, volume 10949, page 109490Y, 2019.

[21] Alan Priester, Shyam Natarajan, Jesse D Le, James Garritano, Bryan Radosavcev, Warren Grundfest, Daniel JA Margolis, Leonard S Marks, and Jiaoti Huang. A system for evaluating magnetic resonance imaging of prostate cancer using patient-specific 3 D printed molds. *American Journal of Clinical and Experimental Urology*, 2(2):127–135, July 2014.

[22] A. Priester, H. Wu, P. Khoshnoodi, D. Schneider, Z. Zhang, N. H. Asvadi, A. Sisk, S. Raman, R. Reiter, W. Grundfest, L. S. Marks, and S. Natarajan. Registration Accuracy of Patient-Specific, Three-Dimensional-Printed Prostate Molds for Correlating Pathology With Magnetic Resonance Imaging. *IEEE Transactions on Biomedical Engineering*, 66(1):14–22, January 2019.

[23] Anant Madabhushi and Michael Feldman. Fused Radiology-Pathology Prostate Dataset. *The Cancer Imaging Archive*, 2016.

[24] P Choyke, B Turkbey, P Pinto, M Merino, and B Wood. Data From PROSTATE-MRI. *The Cancer Imaging Archive*, 2018.

[25] Jonathan I. Epstein, Michael J. Zelefsky, Daniel D. Sjoberg, Joel B. Nelson, Lars Egevad, Cristina Magi-Galluzzi, Andrew J. Vickers, Anil V. Parwani, Victor E. Reuter, Samson W. Fine, James A. Eastham, Peter Wiklund, Misop Han, Chandana A. Reddy, Jay P. Ciezki, Tommy Nyberg, and Eric A. Klein. A Contemporary Prostate Cancer Grading System: A Validated Alternative to the Gleason Score. *European urology*, 69(3):428–435, March 2016.
